# Compressive Sensing Approach to Harmonics Detection in the Ship Electrical Network †

**DOI:** 10.3390/s20092744

**Published:** 2020-05-11

**Authors:** Beata Palczynska, Romuald Masnicki, Janusz Mindykowski

**Affiliations:** 1 Faculty of Electrical and Control Engineering, Gdansk University of Technology, 11/12 Gabriela Narutowicza Street, 80-233 Gdansk, Poland; beata.palczynska@pg.edu.pl; 2 Department of Marine Electrical Power Engineering, Gdynia Maritime University, Morska 81-87, 81-225 Gdynia, Poland; j.mindykowski@we.umg.edu.pl

**Keywords:** harmonics, compressive sensing, signal reconstruction, discrete Radon transform, sparse signal domain

## Abstract

The contribution of this paper is to show the opportunities for using the compressive sensing (CS) technique for detecting harmonics in a frequency sparse signal. The signal in a ship’s electrical network, polluted by harmonic distortions, can be modeled as a superposition of a small number of sinusoids and the discrete Fourier transform (DFT) basis forms its sparse domain. According to the theory of CS, a signal may be reconstructed from under-sampled incoherent linear measurements. This paper highlights the use of the discrete Radon transform (DRT) techniques in the CS scheme. In the reconstruction algorithm section, a fast algorithm based on the inverse DRT is presented, in which a few randomly sampled projections of the input signal are used to correctly reconstruct the original signal. However, DRT requires a very large set of measurements that can defeat the purpose of compressive data acquisition. To acquire the wideband data below the Nyquist frequency, the K-rank-order filter is applied in the sparse transform domain to extract the most significant components and accelerate the convergence of the solution. While most CS research efforts focus on random Gaussian measurements, the Bernoulli matrix with different values of the probability of ones is applied in the presented algorithm. Preliminary results of numerical simulation confirm the effectiveness of the algorithm used, but also indicate its limitations. A significant advantage of the proposed approach is the speed of analysis, which uses fast Fourier transform (FFT) and inverse FFT (IFFT) algorithms widely available in programming environments. Moreover, the data processing algorithm is quite simple, and therefore memory usage and burden of the data processing load are relatively low.

## 1. Introduction

The electric power quality (PQ) in a ship system is described by the set of parameters characterizing a process of generation, distribution, and utilization of electrical energy in all operation states of the ship (maneuvering, sea voyage, staying in port) and its impact on the operation and safety of the ship as a whole. This set of parameters under consideration covers two aspects [1]:

- parameters describing the risk of loss of power supply continuity, and
- parameters of voltage and currents at all the points of the analyzed system.

**Figure 1:**
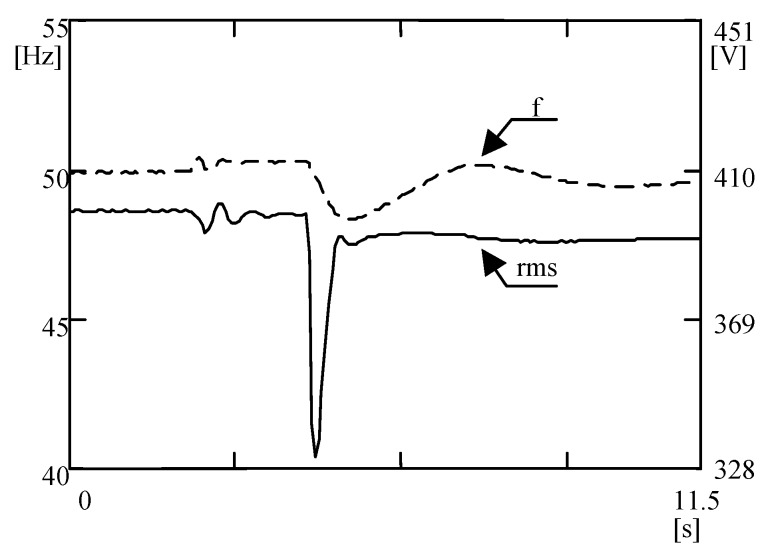
Exemplary voltage and frequency deviations on a ro-ro ship over changing of power plant configuration [3].

**Figure 2:**
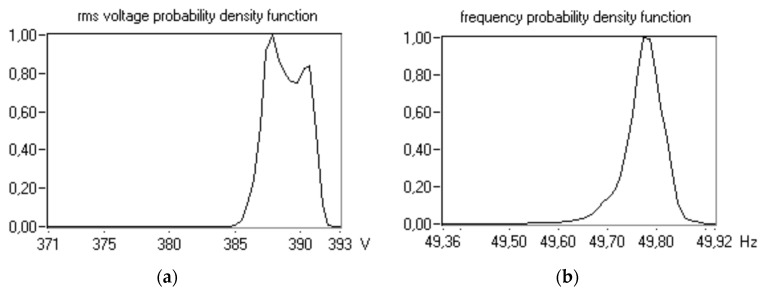
The probability density functions of (**a**) rms voltage and (**b**) frequency in a ferry’s 380 V network during maneuvering [4].

**Figure 3:**
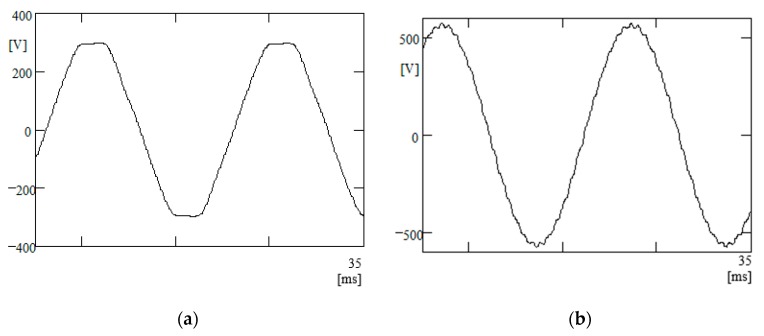
The voltage waveforms in (**a**) 220 V ferry network and (**b**) 400 V network supplied by a shaft generator on a ro-ro ship [3].

Parameters of the first group are essential, but the second group of parameters is significantly better recognized in the area under consideration. Nevertheless, electrical energy must first and foremost be delivered to the consumers, and then its parameters can be evaluated. Bearing in mind the aforementioned assumption, parameters of the first group are mainly associated with correct distribution of active and reactive loads among generating sets working in parallel. A main goal of their control is to avoid the “black-out” phenomenon, resulting from apparent overloading of the ship power station. Parameters of the second group are mainly expressed by the coefficients of rms (root mean square) voltage value and its frequency deviations, coefficients of voltage asymmetry, and coefficients characterizing the shape of voltage and current waveforms, which characterize the distortion of supply voltage from the sinusoidal wave. There are phenomena occurring in ship electrical power systems that can barely be detected by measuring devices in current usage. The suitable estimation of the properties of voltage in the system under consideration requires a wide range of data of steady- and non-steady-state disturbances. The occurrence of various kind of interference is strictly related to different stages of the ship’s exploitation. The variations of rms value of voltage and its frequency over switching from shaft generator to diesel propelled generator on a ro-ro ship (a roll-on/roll-off ship used to carry wheeled cargo) are depicted in Figure 1. For example, Table 1 presents the results of statistical analysis of parameters obtained from measurements on a ferry during maneuvering, in a network with a nominal voltage of 380 V (instantaneous and rms voltage values were obtained for a time interval of 2048 samples within 40 min). The corresponding probability density functions of assuming instantaneous values are shown in Figure 2. In addition to non-steady phenomena, there are steady-state phenomena such as harmonics (Figure 3). The usually description of harmonic distortion is done by means of total harmonic distortion (THD) or/and factors of respective harmonics content (Table 2). Therefore, tests are needed for a relatively long period of the ship’s exploitation processes in various electrical power plant configurations. According to the recently updated International Association of Classification Societies (IACS) requirements [2], newly built ships are to be equipped with devices to continuously monitor the levels of harmonic distortion, while the PQ factors should be measured on existing ships annually under seagoing conditions. The related measurements, therefore, result in the need to collect a large amount of digital data, which in turn leads to a huge memory occupancy and burden in data processing.

A promising solution could be the implementation of a compressing sensing (CS) technique for data acquisition, and further, the use of appropriate algorithms for data reconstruction.

The main idea of CS is to combine sampling and compression of the signals that are sparse or compressible, either in their original domain or in a certain transformed domain. Differently from the typical approach, a new CS technique provides an estimate of the examined signal from a small number of linear incoherent measurements [5,6,7,8]. The linear projection values of the original signal are acquired by a measurement matrix directly with a lower sampling rate than the Nyquist frequency. The sampling frequency does not depend on the signal bandwidth, but on the structure and contents of the information in the signal. The essential assumption in the CS approach is that most of the signals in real applications have a sparse representation in a transform domain, which means a few coefficients are significant, while the rest are negligible or zero. Many of the signals appearing in real applications have a sparse representation in the discrete Fourier transform (DFT) domain [9,10,11,12]. Another relevant condition of the CS technique is the incoherence between measurement (observation) basis and domain in which the signal has a sparse representation. 

Recent research on the CS technique indicates the possibility of accurate signal frequency analysis [13,14]. CS is widely used in diverse fields, e.g., biomedical applications, communication systems, and pattern recognition, as well as electrical PQ estimation [15,16]. Various new techniques for identifying and estimating harmonic sources in electricity supply systems have been presented in the literature [17,18,19,20,21,22,23,24,25,26,27,28]. The papers [17,18,19,20,21] concern the distributed monitoring of harmonic and interharmonic pollution in electrical power delivery systems. In order to reduce implementation costs, the authors propose a distributed architecture, based on cost-effective nodes that use the CS strategy. Some papers [22,23,24,25,26,27,28] consider the different methods of compressive sampling and innovative CS algorithms for reconstruction of the PQ interference signal.

There are many CS reconstruction algorithms in the literature that present different approaches for finding a sparse estimation of the original input signal, based on a minimum number of measurements, and characterized by interference resistance, speed, complexity, good performance, etc. One type of reconstruction method is based on the Bayesian framework, where the posterior density function of the sparse solution is estimated [29]. This approach to CS constitutes the reconstruction as a Bayesian inference problem and is applicable for the input signals that fit some known probability distribution. Bayesian compressive sensing (BCS) can be used to estimate the time–frequency spectrum of a nonstationary signal [30]. A certain type of BCS approach is the Monte Carlo Bayesian compressive sensing (MC-BCS) method, which is distinguished by the fact that it numerically evaluates the posterior sparse solution [31].

In many cases, reconstruction algorithms perform moderately at low measurement rates and are computationally expensive. In practice, the purpose of measurement is not always to perfectly reconstruct the input signal, but to determine some of its parameters. Recent advances in the areas of CS have shown that effective inference is possible directly from the compressive measurements, without reconstruction, using correlational features [32]. This idea is currently developed in computational imaging [33,34,35]. A good research direction might be to consider the possibility of using this approach for harmonic detection. However, the contribution of the present paper is the CS reconstruction algorithm, which allows obtaining a satisfactory accuracy of harmonic detection based on a much smaller number of measurements than in the classic approach.

This paper proposes the application of a fast reconstruction procedure based on the CS technique for detecting harmonics in a tested signal. The procedure uses random projections as measurements. The measurement matrix, generated from Bernoulli’s random variables, allows recovering the signal with high accuracy. The ℓ1-minimization problem in the CS signal reconstruction is solved by means of discrete Radon transform (DRT) techniques with the use of the K-rank-order filter in the signal’s sparse domain to accelerate the solution convergence.

The organization of the remainder of this paper is as follows. Section 2 discusses the CS framework in three aspects: the sparsity of the signal, the sensing process, and the reconstruction condition. Section 3 explains the algorithmic implementation of the reconstruction procedure based on DRT techniques. Section 4 shows the preliminary results of simulation obtained for the selected multitone signals. A brief discussion is carried out in Section 5. Finally, concluding remarks are formulated in Section 6.

## 2. Compressive Sensing Framework

The idea of CS is to recover high dimension sparse or compressible signals through low dimension measurements. Three main issues form the basis of the CS theory. The first is seeking the domain of sparsity, in which a signal can be represented by a few significant components, compared to the total signal length. Another important issue concerns the design of an appropriate measurement matrix that directly affects the results of the signal reconstruction. The last deals with the implementation of the reconstruction algorithm, which performs the sparse estimation of the original input signal, from compressive measurements.

### 2.1. Sparse Representation

Suppose that the real signal $x\in R^{N}$ has a *K*-sparse representation in an orthonormal transformation basis $\psi \in R^{N\times N}$. This means that the signal can be expanded to *K* nonzero coefficients in basis ψ (*K≪N*). The approximation of *x* can be expressed as follows [5]:

(1) $$x=\sum_{i=1}^{N}a_{i}\cdot \psi_{i}=\psi \cdot a,$$

where $a\in R^{N}$ represents the sparse transform domain coefficients of signal *x*. 

Taking into account a multicomponent signal that consists of *K* sinusoids, it can be described by:

(2) $$x_{n}=\frac{1}{N}\sum_{k=1}^{K}X_{k}\cdot \exp(j\cdot \frac{2\pi }{N}\cdot n\cdot k).$$

When the sinusoids are of infinite extent, Equation (2) presents the K-sparse representation of such a signal in terms of DFT, since:

(3) $$X_{k}=\sum_{n=1}^{N}x_{n}\cdot \exp(-j\cdot \frac{2\pi }{N}\cdot n\cdot k),$$

where *X_k_* is a vector of DFT coefficients wherein *K* coefficients, at most, are nonzero. 

The transformation matrix, ψ, created on the Fourier basis is determined by [9]:

(4) $$\psi_{n,k}=\frac{1}{\sqrt{N}}\exp(j\cdot \frac{2\pi }{N}\cdot n\cdot k).$$

In this paper, the transformation domain is defined directly by the discrete Fourier transform.

### 2.2. Sensing Process

The measurement process is modelled by projections of the signal *x* onto vectors $\left\{\varphi_{1},\cdots ,\varphi_{M}\right\}$ forming the measurement matrix $\varphi \in R^{M\times N}$. The vector of acquired samples $y\in R^{M}$ is defined as [5]:

(5) $$y=\left[\begin{array}{c} y_{1}\\ \vdots \\ y_{M} \end{array}\right]=\left[\begin{array}{ccc} \varphi_{11} & \cdots & \varphi_{1N}\\ \vdots & \ddots & \vdots \\ \varphi_{M1} & \cdots & \varphi_{MN} \end{array}\right]\cdot \left[\begin{array}{c} \begin{array}{l} x_{1} \end{array}\\ \vdots \\ \begin{array}{l} x_{N} \end{array} \end{array}\right]=\varphi \cdot x.$$

The number of measurements *M* is much smaller than the length of the input signal *N*, which at the same time is greater than the level of sparsity *K*. The transformation matrix ψ (related to the sparsity of the signal) and the measurement matrix φ (used in the measurement procedure) must be incoherent to ensure adequate reconstruction. The coherence measure is described by [7]:

(6) $$\mu (\varphi ,\psi )=\sqrt{N}\cdot \underset{0\leq i,j\leq N}{\max}\left|\langle \varphi_{i},\psi_{j}\rangle \right|.$$

The coherence takes values from the interval [7]:

(7) $$1\leq \mu (\varphi ,\psi )\leq \sqrt{N}.$$

The coherence should be as small as possible. 

In the paper, the random Bernoulli matrix as the measurement matrix is used to ensure the incoherence of bases [36,37]. It is created by a pseudorandom pattern of ones and zeros that guarantees the Bernoulli distribution, described by the probability density function, over possible outcomes *k*:

(8) $$f(k;p)=p^{k}{\left(1-p\right)}^{1-k}\;\;\;\;for\;\;\;\;k\in \left\{0,1\right\},$$

where *p* is the ones probability of Bernoulli distribution, 0≤p≤1.

Then, the acquisition process is described by following expression [36]:

(9) $$y=\langle \varphi ,x\rangle =\sum_{j=0}^{N-1}\varphi_{i,j}\cdot x_{j},$$

where $\varphi_{i,j}$ is the (*i*, *j*)th entry of the random binary matrix φ.

Taking into account Equation (1), the measurement signal *y* becomes [11]:

(10) y=φ·ψ·a=Θ·a,

where $\Theta \in R^{M\times N}$ is a reconstruction (sensing) matrix, in the form of:

(11) $$\Theta =\left[\begin{array}{ccc} \varphi_{11} & \cdots & \varphi_{1N}\\ \vdots & \ddots & \vdots \\ \varphi_{M1} & \cdots & \varphi_{MN} \end{array}\right]\cdot \left[\begin{array}{ccc} \begin{array}{l} \psi_{11} \end{array} & \cdots & \begin{array}{l} \psi_{1N} \end{array}\\ \vdots & \ddots & \vdots \\ \begin{array}{l} \psi_{M1} \end{array} & \cdots & \begin{array}{l} \psi_{MN} \end{array} \end{array}\right]=\left[\begin{array}{ccc} 0 & \cdots & 1\\ \vdots & \ddots & \vdots \\ 1 & \cdots & 1 \end{array}\right]\cdot \left[\begin{array}{ccc} \begin{array}{l} \psi_{11} \end{array} & \cdots & \begin{array}{l} \psi_{1N} \end{array}\\ \vdots & \ddots & \vdots \\ \begin{array}{l} \psi_{M1} \end{array} & \cdots & \begin{array}{l} \psi_{MN} \end{array} \end{array}\right].$$

Finally, the sensing matrix represents a partial random inverse Fourier transform matrix obtained by omitting rows from the transformation matrix ψ, which corresponds to unavailable sample positions. In this case, the relation of coherence *µ*, with number of measurements *M* is given by [13]:

(12) $$M\geq c\mu K\log\frac{N}{K},$$

This expression reveals that a lower coherence value is desirable, resulting in fewer measurements required for the CS reconstruction.

### 2.3. Reconstruction Condition

To ensure reconstruction of the sparse signal *x* from compressive measurements *y*, the inverse problem of Equation (5) should be solved, which gives an infinite number of possible solutions. Consequently, optimization algorithms based on the *ℓ*_1_-norm minimization are commonly applied [8]:

(13) $$\hat{a}=\arg\min{\left\|a\right\|}_{1}\;\;\;\;subject\;to\;y=\Theta \cdot a,$$

where $\hat{a}$ denotes the estimate of a and ${\left\|a\right\|}_{1}$ means *ℓ*_1_-norm of a.

According to Equation (13), the estimation of the input signal can be made as [12]:

(14) $$\hat{x}=\varphi \cdot {\left(\Theta^{T}\cdot \Theta \right)}^{-1}\cdot \Theta^{T}\cdot y,$$

where ($\Theta^{T}\cdot \Theta$)^−1^ is the pseudoinverse matrix of product of matrices.

## 3. Reconstruction Algorithm

Searching for a solution to Equation (14), one should solve a set of equations with a non-quadratic system matrix. The solution of the normal Equation (14) can be interpreted in formalism of the direct reconstruction method based on discrete Radon transform (DRT) and inversion of the discrete Radon transform (IDRT) techniques [38,39,40]. Using the Radon transform scheme along with the matrix formalism implies ordinary matrix operations have equivalent Radon transform operations.

### 3.1. Relationship between Matrix Operations and the Radon Transform

The Radon transform *R*(*p,q*) of two-dimensional function *g*(*x,y*) is found by stacking or integrating values of *g* along slanted lines. The location of the line is determined from the line parameters slope, *p*, and line offset, *τ* [38].

(15) $$R(p,q)[g(x,y)]=\int_{-\infty }^{\infty }g(x,\tau +px)dx.$$

By sampling four variables [38]:

(16) $$\begin{array}{l} x=x_{m}=x_{\min}+m\Delta x,\;\;m=0,1,\ldots ,M-1\\ y=y_{n}=y_{\min}+n\Delta y,\;\;n=0,1,\ldots ,N-1\\ p=p_{k}=p_{\min}+k\Delta p,\;\;k=0,1,\ldots ,K-1\\ \tau =\tau_{h}=\tau_{\min}+h\Delta \tau ,\;\;h=0,1,\ldots ,H-1 \end{array}$$

where *x*_min_, *y*_min_, *p*_min_, *τ*_min_ represents the position of the first sample, and Δ*x*, Δ*y*, Δ*p*, Δ*τ* represents the sampling distance.

The Radon transform can be approximated by the DRT using a nearest neighbor approximation in the *y* direction, hence [38]:

(17) $$\overset{◡}{g}(k,h)=\Delta x\sum_{m=0}^{M-1}g(m,n(m;k,h))$$

in which [38]:

(18) $$n(m;k,h)=\left[\frac{p_{k}x_{m}+\tau_{h}-y_{\min}}{\Delta y}\right],$$

wherein the expression in [ ] means rounding the argument to nearest integer.

Suppose the matrix $\overset{◡}{g}(k,h)$ is converted to a vector [38]:

(19) $$b_{i}=b_{kH+h}=\overset{◡}{g}(k,h),$$

And

(20) $$x_{j}=x_{nM+m}=g(m,n),$$

the Radon transform can be written in the form [38]:

(21) b=Ax,

where $A\in R^{I\times J}$ is the non-quadratic transformation matrix, and *I* = *KH*, *J* = *MN*.

The solution of Equation (21) can be interpreted in the formalism of the direct reconstruction methods:

(22) $$x={\left(A^{T}A\right)}^{-1}A^{T}b.$$

There are several reconstruction schemes for the inversion of DRT based on linear algebra, such as iterative methods, e.g., the algebraic reconstruction technique (ART), the conjugate gradient (CG) algorithm, and the expectation maximization (EM) algorithm. On the other hand, it uses the Fourier slice theorem, which allows the Fourier transform of the projection data to be considered as discrete samples of the object in the Fourier domain. In this case, the sensing matrix (Equation (11)) can be considered an under-sampled discrete Fourier operator [40]. 

### 3.2. Radon’s Inversion Formula

Considering the sensing acquisition process described in Equations (9) and (10), as standard projections onto single scalar measurements, to recover the input signal *x* the maximum likelihood reconstruction method with EM algorithm is applied [38]. The maximum likelihood estimator of *x* based on a random sample is the sample mean, so the reconstruction formula is defined as [41]:

(23) $$\bar{x}=\frac{1}{1+i}\sum_{j=0}^{i-1}\frac{1}{{\mathrm{var}}_{j}}y_{j}\cdot \varphi_{j},$$

where var means a variance of random distribution, for Bernoulli distribution ${\mathrm{var}}_{j}=p_{j}(1-p_{j})$.

The algorithm works in a loop, and in each iteration, it checks whether Equation (23) converges. The processing loop stops when the threshold *t* meets the given condition [41]:

(24) $$0\ll t\leq \frac{\left|y_{i}\right|}{\left|y_{i}\right|+\left|y_{i}-\langle \varphi_{i},{\hat{x}}_{i}\rangle \right|}\leq 1,$$

where ${\hat{x}}_{i}$ is the estimate of *x_i_* at the *i*th iteration, defined as [41]:

(25) $${\hat{x}}_{i}=F^{-1}\left\{Rank\left(F\left\{\bar{x}\right\}\right)\right\},$$

where *Rank* (*) symbolizes a *K*-rank-order filter.

The *K*-rank-order filter is applied in the Fourier domain to accelerate the convergence. The filter operates in the following way. Let’s consider an input vector $X_{k}=F\left\{\bar{x}\right\}=\left[X_{1},\cdots ,X_{N}\right]$. First, the filter sorts the input array with the values arranged in descending order. Then, it extracts the *K* most significant components from the input vector and assigns zeros to the remaining places. As a result, the computational burden of the inverse Fourier transform, performed in the next step, is reduced.

## 4. Numerical Simulations

**Figure 4:**
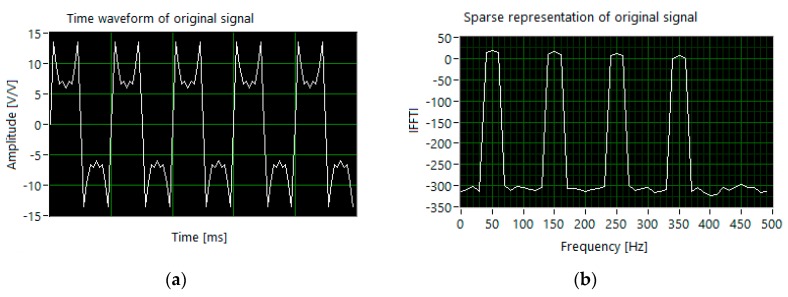
The results of simulation for set 1: (**a**) the segment of time waveform; (**b**) discrete Fourier transform (DFT) components of input signal.

The simulations were performed using a virtual instrument designed based on an accessible application in the LabVIEW environment [32]. As an example, a multi-tone signal with fundamental harmonic 50 Hz was simulated, according to the parameter sets shown in Table 3. In presented exemplary results of simulations, sparsity level *K* was set to 7. The sampling frequency was equal to 10 kHz and the length of the time window was equal to 1000 samples. The time waveform and sparse representation of the tested signal in the Fourier domain is presented in Figure 4.

**Figure 5:**
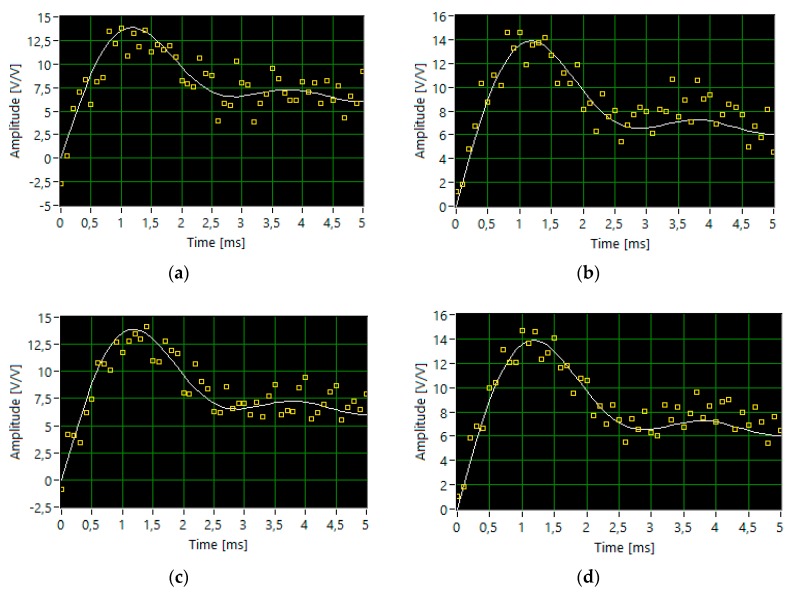

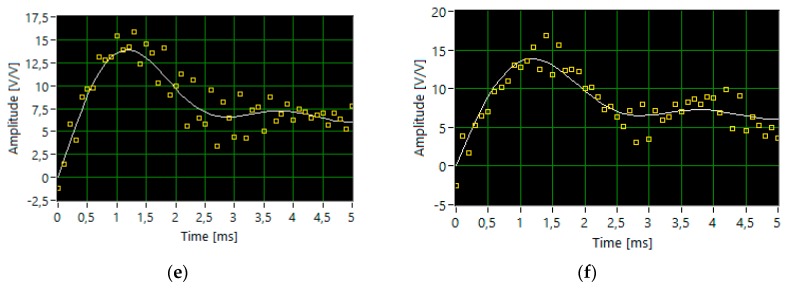
The segments of time waveforms of the original (white line) and reconstructed signal (dotted yellow line) for following parameters: *p*, accuracy and the number of iterations, respectively: (**a**) 0.01, 99.67%, 308; (**b**) 0.1; 99.56%, 330; (**c**) 0.3, 99.76%, 490; (**d**) 0.5, 99.99%, 690; (**e**) 0.8, 99.9%, 406; (**f**) 0.9, 99.39%, 384.

First, the influence of the value of the ones probability *p* on the level of quality of a signal reconstruction and the number of iterations (measurements) was examined. For a fixed *t*-threshold level of 99%, the value *p* was changed in the range of 0.01 to 0.9 (Figure 5). The simulation results show that depending on the value of *p* the algorithm converges for a different number of iterations. With an assumed *t*-threshold, the loop stops more quickly at the lowest *p*-values, with an approximate accuracy of 99.67%. The algorithm achieves the best reconstruction accuracy for a *p*-value of 0.5 but with the largest number of measurements. Comparing the segments of time waveforms of the original signal and reconstructed signal (Figure 5) for the parameters probability *p*, accuracy, and the number of iterations, we can preliminarily conclude that the most optimal case occurs at *p* = 0.3, for which, at 490 iterations, the accuracy is in the order of 99.8%.

**Figure 6:**
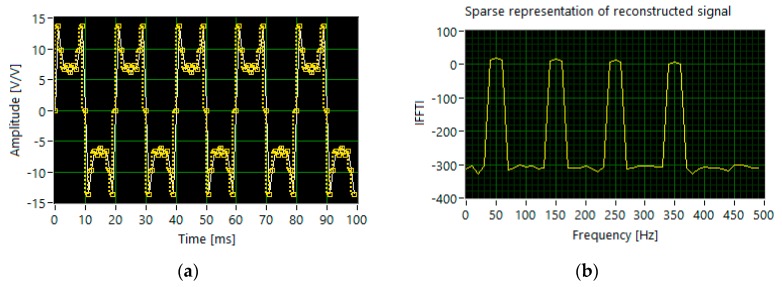
The results of simulation for set 1: (**a**) the segments of time waveforms of the original (white line) and reconstructed signal (dotted yellow line); (**b**) DFT components of the reconstructed signal.

In the case of generating the tones with the slightly different levels of amplitude (set 1), the algorithm identifies harmonics with accuracy above 97%. The most accurate signal reconstruction (99.32%) is obtained for 210 sampled measurement signals, acquired with the ones probability *p* equal to 0.3 (Figure 6).

**Figure 7:**
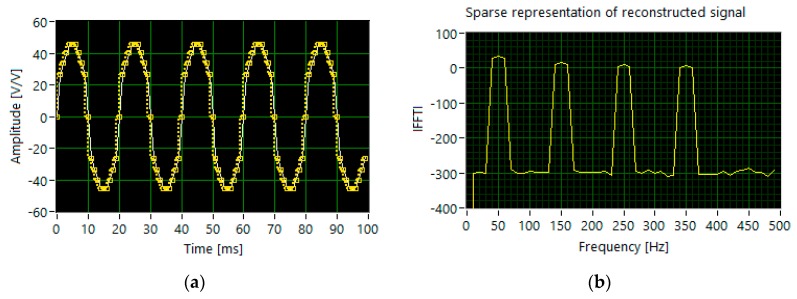
The results of simulation for set 2: (**a**) the segments of time waveforms of the original (white line) and reconstructed signal (dotted yellow line); (**b**) DFT components of the reconstructed signal.

For the multi-tone signal with different levels of amplitude (set 2), the reconstruction based on 280 measurements and the same features of the measurement matrix does not allow the correct detection of all harmonics (Figure 7).

**Figure 8:**
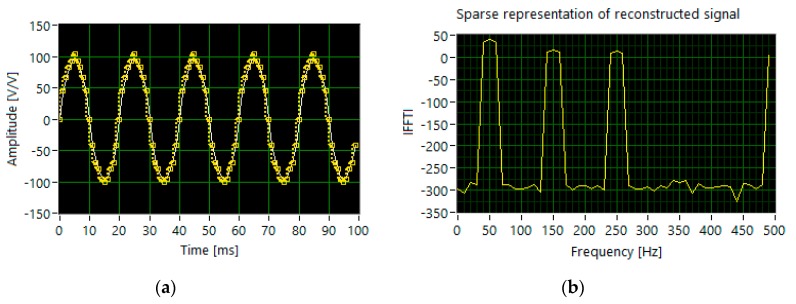
The results of simulation for set 3: (**a**) the segments of time waveforms of the original (white line) and reconstructed signal (dotted yellow line); (**b**) DFT components of the reconstructed signal.

The proper reconstruction requires twice as many measurements, i.e., 400. The scenario with the dominant fundamental harmonic (set 3) shows that 500 sampled measurements are not sufficient to identify all components in the frequency domain (Figure 8). The algorithm incorrectly detects 7th harmonics. Analyzing the results of simulations carried out for set1, set2 and set3, respectively, shown in Figure 6, Figure 7 and Figure 8, and bearing in mind the data listed in Table 1, we may conclude that increasing the number of measurements results in better efficiency of harmonic identification. In this case, the number of random samples reaches 800.

**Figure 9:**
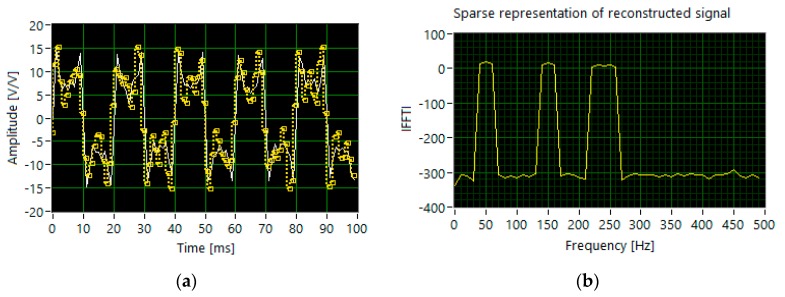
The results of simulation for the input signal (set 1) with the additive noise: (**a**) the segments of time waveforms of the original (white line) and reconstructed signal (dotted yellow line); (**b**) DFT components of the reconstructed signal. The number of measurements is equal to 210.

**Figure 10:**
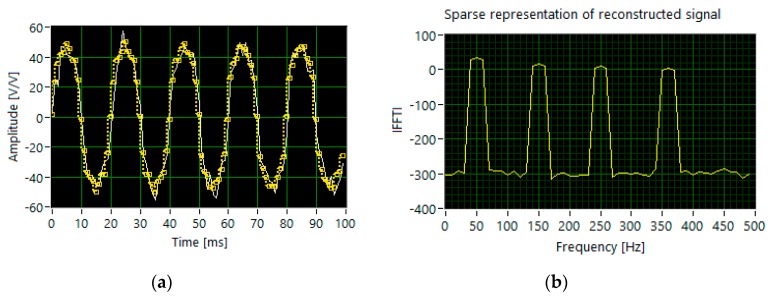
The results of simulation for the input signal (set 2) with the additive noise: (**a**) the segments of time waveforms of the original (white line) and reconstructed signal (dotted yellow line); (**b**) DFT components of the reconstructed signal. The number of measurements is equal to 400.

To study the effect of noise on the efficiency of signal reconstruction, white Gaussian noise is added to obtain a signal to noise ratio (S/N) of 20 dB. Figure 9 shows the result of a sparse reconstruction of a multi-tone waveform with insignificant differences of amplitude levels (set 1) for 210 iterations (measurements). The presence of noise adversely affects the correct identification of harmonics. In the case of set 2, in which one tone is characterized by a much higher amplitude level, adding noise does not affect the detection of harmonics (Figure 10). Noise interference distorts the precision of the spectral analysis of a multi-tone signal when there is a strong dominant component in the signal (set 3).

**Figure 11:**
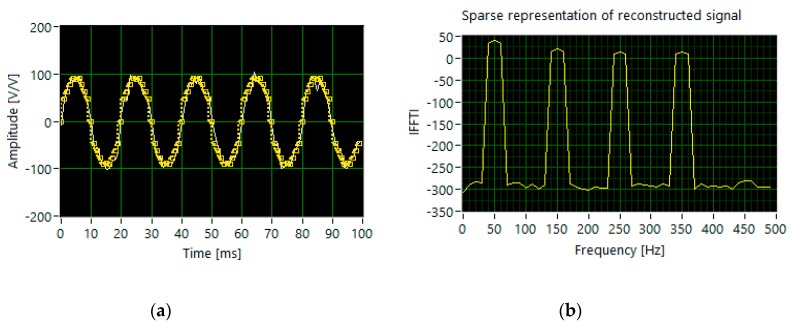
The results of simulation for the input signal (set 3) with the additive noise: (**a**) the segments of time waveforms of the original (white line) and reconstructed signal (dotted yellow line); (**b**) DFT components of the reconstructed signal. The number of measurements is equal to 788.

Performing a spare signal reconstruction allows for more accurate detection of harmonics in the signal (Figure 11). Taking into account the results of simulation with the additive noise corresponding to the appropriate data sets shown in Figure 9, Figure 10 and Figure 11, we may formulate a preliminary opinion that accurate detection of harmonics is only possible with a very large number of iterations in the measurement algorithm, but the number of measurements is still smaller than when using the classic approach.

## 5. Discussion

### 5.1. Reconstruction Accuracy 

For the reconstruction of one-dimensional sparse signals, the mean square error (MSE) is adopted as the reconstruction performance metric:

(26) $$MSE=Ε\left[{\left\|\hat{x}-x\right\|}_{2}^{2}\right].$$

**Figure 12:**
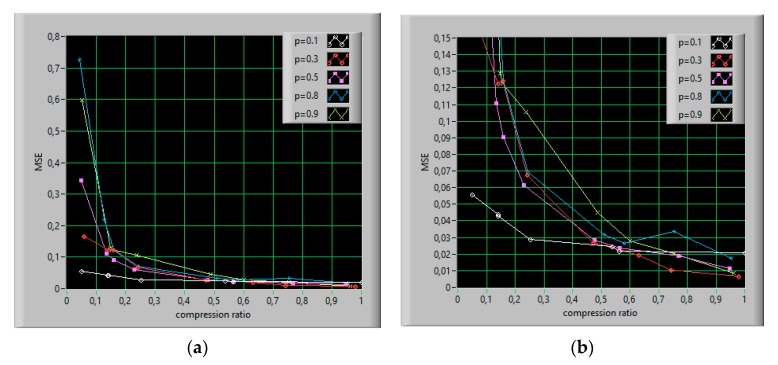
Mean square error (MSE) versus compression ratio for different values of the ones probability *p*, (**a**) in a full range, (**b**) for a threshold of MSE = 0.15.

As shown in Figure 12, the MSE values depend on the compression ratio (M/N), and therefore on the number of measurements obtained. In addition, the effect of the actual distribution of randomly taken samples on the quality of the reconstruction can be seen. Suppose MSE = 0.15 as a threshold to distinguish between successful and unsuccessful reconstruction. It can be noted that most values of the ones probability *p* below 0.1 do not allow effective signal reconstruction (Figure 12b). In the presented simulations, the compression ratio equal to 0.1 means that for the input signal length of 1000 samples, 100 iterations should be performed. The measurement algorithm determines the condition of stopping the processing loop (Equation (24)), which refers to the accuracy of the input signal recovery. For small *p*-values, the algorithm converges quickly, resulting in less than 80% accuracy.

### 5.2. Frequency Detection Accuracy

**Figure 13:**
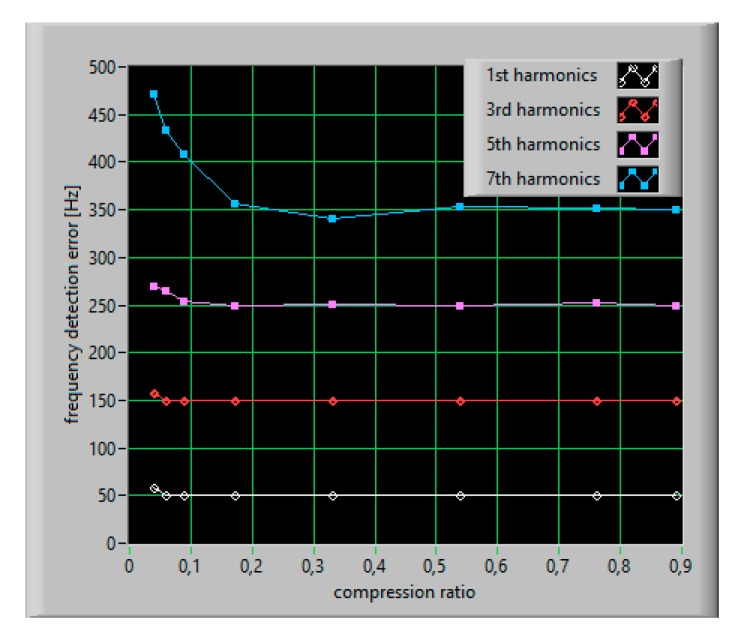
The accuracy of frequency detection versus compression ratio.

The main issue for spectral detection of a multi-toned waveform is to distinguish the individual sinusoidal component in the frequency domain. In the DFT algorithm, coefficients obtained from a sequence of *N* samples, taken at sampling interval Δ*T* = 1*/f_s_*, are determined on a discrete frequency grid whose step size Δ*f* is equal to 1/*N*Δ*T*. This determines the minimum theoretical frequency separation at which two frequency components can be determined. For example, a 5 Hz frequency resolution is required to evaluate THD or harmonic contents in the ship’s electrical network. The suggested observation time is 200 ms (equal to 10*T*_0_ for 50 Hz systems or 12*T*_0_ for 60 Hz systems). In the CS-DFT approach, only *M* < *N* samples of the input signal are taken but they should represent 10 periods of the input signal to ensure the required resolution of the analysis. As shown in Figure 13, the accuracy of frequency detection for lower harmonics practically does not depend on the compression ratio for its value above 0.1. However, both in the case of reconstruction accuracy and in a case of frequency detection accuracy, the minimum number of measurements (minimum value of the compression ratio) can be specified for which it is possible to correctly detect frequency components. An effective detection of higher harmonics, for a fixed frequency resolution of analysis, requires a greater number of iterations.

### 5.3. The Computation Burden

**Figure 14:**
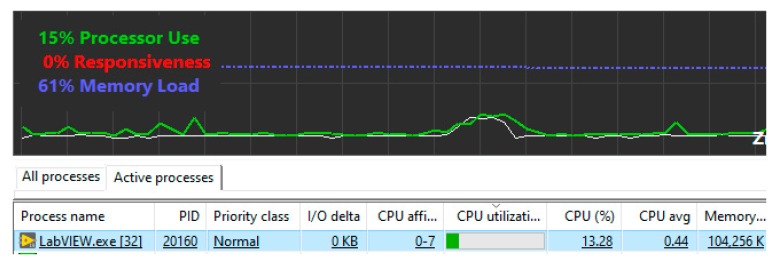
The graphical user interface of Lasso software.

The code was executed on an Intel (R) Core (T) i7-2600 CPU @ 3.4 GHz processor with 8 GB RAM. The CPU load during the running algorithm was estimated with software Process Lasso [42] (Figure 14). The computer burden did not exceed 15%. 

**Figure 15:**
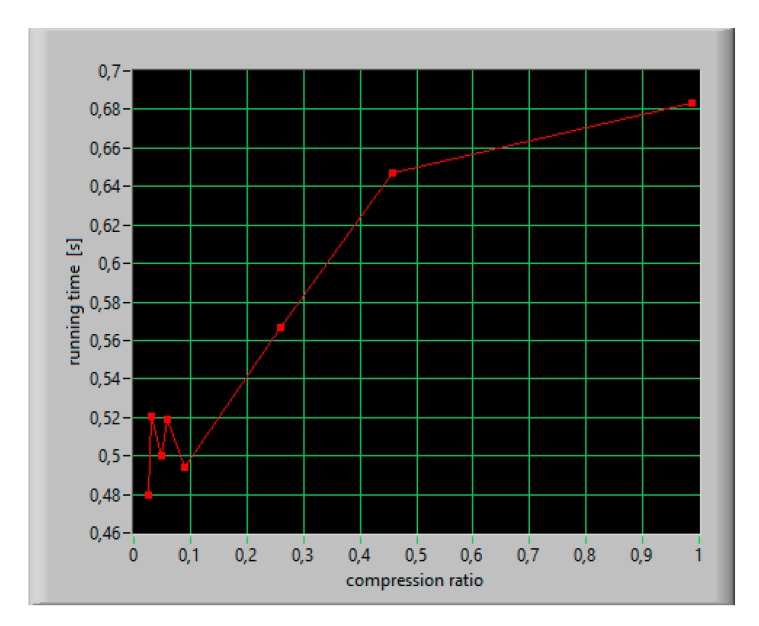
The running time versus compression ratio.

The running time of a reconstruction algorithm was appointed based on 100 trials for different numbers of iterations, which means different reconstruction error. For compression ratios in the range from 0.1 to 0.2, the performance time of the algorithm was about 520 ms (Figure 15). In Table 4, several algorithms are compared for computational recovery time required for Fourier-based CS measurements [27,43]. CS convex optimization algorithms and greedy algorithms are considered for reconstruction accuracy equal to 80%. Taking into account the processing time, the presented algorithm runs much faster than optimization algorithms but slightly slower than greedy algorithms.

The preliminary results of numerical simulations performed using the fast reconstruction algorithm show the limitations of effective reconstruction based on the CS method. The reconstruction accuracy is good in the case of multi-tone signals consisting of components whose amplitude levels do not differ significantly. The application of the presented reconstruction algorithm to the signal, in which the dominant harmonic occurs, requires the pre-conditioning of the input signal. This consists of the rejection of the fundamental component from the examined waveform. The accuracy of the reconstruction is also influenced by the properties of the random measurement matrix. While most studies to date have focused on Gaussian random measurements, this paper investigated the performance of a matrix with a Bernoulli distribution. The Bernoulli matrix with different values of the ones probability *p* was used in the presented simulations. Small *p*-values, e.g., equal to 0.1, means the probability when each element of the measurement matrix has a 10% chance of being one and a 90% chance of being zero. A lower value of the *p* parameter of the Bernoulli distribution results in a sparser solution. At the same time, the number of iterations (measurements) is reduced, which results in a less-accurate reconstruction. The optimal *p*-value of 0.3 was determined, based on the simulations carried out.

## 6. Conclusions

In ship operations, effective resource management is crucial to its proper functioning. In view of the multitude of measurements on a ship, the search for effective means for their implementation is of great importance. One approach to reduce the load on memory and data processing systems is to use the CS technique.

Preliminary results of the simulations carried out confirmed that the key advantage of the proposed approach is the high speed of analysis, which uses software algorithms widely available in programming environments. Furthermore, the data processing algorithm is quite simple, and therefore memory consumption and burden in data processing are relatively low.

The future task of the planned research is to improve and develop the procedures presented with an extension to a real object investigation in a ship environment.

## Figures and Tables

**Table 1:** The statistical properties of rms voltage and frequency in the network of a ferry [4].

Statistics	RMS Voltage (V)	Frequency (Hz)
mean	389.097	49.778
standard deviation	2.337	0.044
mode	382.39	49.78

**Table 2:** Exemplary harmonic contents of analysed signals [3].

Harmonic Order	Harmonic Contents in Signal from Figure 3a (%)	Harmonic Contents in Signal from Figure 3b (%)
2	0.2	0.0
3	0.1	0.0
5	2.9	0.1
7	1.7	0.3
11	0.2	0.1
13	0.1	0.2
23	0.0	0.5
25	0.0	1.6
THD	3.4	1.8

**Table 3:** The parameters of the input signals.

Harmonic Order	Amplitude (V/V)		
	Set 1	Set2	Set 3
1	10	50	100
3	6	6	6
5	4	4	4
7	2	2	2

**Table 4:** Average running time of reconstruction algorithms [27,43].

Name of Algorithm	K Sparsity	Run Time (s)
*ℓ*_1_-Least Squares (L1-LS) Regularization Algorithm ^1^	-	14.37
Fixed Point Continuation (FPC) Method ^1^	-	8.66
Orthogonal Matching Pursuit (OMP) ^2^	10	0.19
Compressive sampling matching pursuit (CoSaMP) ^2^	10	0.28
Presented in the paper	7	0.54

^1^*ℓ*_1_-norm -fast algorithm, ^2^ greedy algorithm.

## Abbreviations

The following abbreviations are used in this manuscript:

CS	Compressive Sensing
PQ	Power Quality
IACS	International Association of Classification Societies
DFT	Discrete Fourier transform
FFT	Fast Fourier Transform
DRT	Discrete Radon Transform
IDRT	Inversion of the Discrete Radon Transform

## Nomenclature

$x\in R^{N}$	real signal
*N*	length of signal
*M*	number of compressive measurements
*K*	size of sparsity
$\psi \in R^{N\times N}$	transformation matrix
$a\in R^{N}$	sparse transform domain coefficients
*X_k_*	vector of DFT coefficients
$\varphi \in R^{M\times N}$	measurement matrix
$y\in R^{M}$	vector of acquired samples
μ(φ,ψ)	coherence
$\Theta \in R^{M\times N}$	reconstruction (sensing) matrix
$\hat{a}$	estimate of a
${\left\|a\right\|}_{1}$	*ℓ*_1_ norm of a
*p*	the ones probability of the Bernoulli distribution
